# Evaluation of the Transverse Carpal Ligament in Carpal Tunnel Syndrome by Shear Wave Elastography: A Non-Invasive Approach of Diagnosis and Management

**DOI:** 10.3389/fneur.2022.901104

**Published:** 2022-07-01

**Authors:** Huaiyu Wu, Keen Yang, Xin Chang, Zhaokang Liu, Zhimin Ding, Weiyu Liang, Jinfeng Xu, Fajin Dong

**Affiliations:** ^1^Department of Ultrasound, Shenzhen People's Hospital, First Clinical College of Jinan University, Second Clinical College of Jinan University, First Affiliated Hospital of Southern University of Science and Technology, Shenzhen, China; ^2^Department of Neurology, Shenzhen People's Hospital, First Clinical College of Jinan University, Second Clinical College of Jinan University, First Affiliated Hospital of Southern University of Science and Technology, Shenzhen, China; ^3^Department of Hand and Micro-Vascular Surgery, Shenzhen People's Hospital, First Clinical College of Jinan University, Second Clinical College of Jinan University, First Affiliated Hospital of Southern University of Science and Technology, Shenzhen, China

**Keywords:** carpal tunnel syndrome, transverse carpal ligament, median nerve, ultrasound, shear wave elastography, nerve conduction study

## Abstract

**Objectives:**

The goal of this work is to determine the clinical value of the transverse carpal ligament (TCL) in carpal tunnel syndrome (CTS) for guiding subsequent treatment.

**Methods:**

This study analyzed patients who underwent median nerve (MN) ultrasound (US) examination of the wrist from April 2020 to April 2021. The cross-sectional area and anteroposterior diameter of the MN, as well as the TCL thickness and stiffness, were measured from images. The intra-group and intra-patient subgroup differences were compared using a *t-*test and a rank test. We also utilized receiver operating characteristic (ROC) curves to diagnose CTS and evaluate the severity.

**Results:**

The final cohort consisted of 120 wrists (bilateral) from 60 samples, evenly balanced across the patient and control groups according to their CTS diagnosis. In the unilateral positive patient subgroup, the MN and TCL of the positive hand were significantly thicker and stiffer than the negative counterparts (both, *p* < 0.05). The values from the right were also thicker and stiffer than the left (both, *p* < 0.05) in patients with bilateral CTS. The MN and TCL of the patient group were also significantly thicker and stiffer than those of the control group (both, *p* < 0.001). For diagnosing CTS, the area under the curve (AUC) of TCL thickness and stiffness at the distal carpal tunnel (DCT) ranged between 0.925 and 0.967. For evaluating CTS severity, we found that the optimal TCL stiffness is sufficient for diagnosing mild and non-mild patient cases (AUC: Emean = 0.757, Emax = 0.779).

**Conclusions:**

Shear wave elastography is therefore an effective method for CTS diagnosis and management.

## Introduction

When the median nerve (MN) is compressed in the wrist, it causes numbness and weakness, which lead to a chronic condition called carpal tunnel syndrome (CTS) (1). The common causes of MN compression include the thickening of the transverse carpal ligament (TCL), tenosynovitis, synovitis, etc. (2, 3), and compression by the TCL is the most prevalent cause. Therefore, understanding the situation of TCL and identifying the severity of MN are crucial pieces of information that can guide subsequent treatment.

The nerve conduction study (NCS) is used to assess peripheral nerve injury by measuring changes in nerve currents for diagnosing CTS and evaluating the severity of the associated neuropathy (4). Nevertheless, others consider the NCS to be an “unnecessary evil” since it is an invasive operation requiring multiple shocks and a high 20% false positive rate (5). Moreover, NCS cannot directly observe either the anatomical changes of the nerve or the compression etiologies (6).

Grayscale ultrasound (US) and shear wave elastography (SWE) are non-invasive imaging modalities that can complement NCS in the diagnosis of CTS (7–11). Grayscale US can not only directly observe changes to the MN by simply measuring the cross-sectional area (CSA), but can also detect the cause of compression (7, 8). In our previous work that confirmed CTS by surgical intervention, we found that in grayscale images, the MN was not always obviously compressed but rather swollen or even of normal condition, which is indistinguishable from the MN dysfunction at the TCL, a median neuropathy at the wrist (MNW) that can occur independently with CTS (10). In addition, both the MN stiffness of the CTS or MNW increased when using SWE (11, 12). Therefore, using CSA_MN_ or MN stiffness does not easily distinguish between CTS and MNW.

At present, a few studies have investigated the elasticity of the TCL of normal people (13, 14) and CTS using acoustic radiation force impulse (ARFI) and strain elastography (15–17). The results showed that the thickness and stiffness of TCL are significantly increased in patients with CTS compared with healthy controls. Despite these promising results, the size of the participating cohort in these studies was small and the relationship between TCL and NCS was not explored.

To address this gap in the field, the goal of our study is to use grayscale US and SWE to compare the thickness and stiffness of the TCL, the CSA_MN_, and the anteroposterior diameter of the MN (AP_MN_) in healthy controls and patients with CTS. In addition, we investigated the TCL thickness and stiffness in relation to NCS severity. By addressing these research questions, we hope to provide novel insight into the TCL in CTS that can help clinicians to guide subsequent treatments.

## Materials and Methods

### Patient Selection

This retrospective study was approved by the Institutional Review Board of our hospital. All patients provided written informed consent prior to participation. We consecutively enrolled patients who underwent a wrist nerve examination using US between April 2020 and April 2021. The enrolled participants were assigned to either the patient or control group according to their clinical symptoms. Specifically, participants in the patient group exhibited symptoms of median neuropathy and clinically suspected CTS, whereas participants in the control group exhibited no symptoms of neuropathy. Exclusion criteria were applied to both the patient and control groups and included the following: incomplete clinical data and invasive treatment results of the wrist. Inclusion criteria for both the patient and control groups were as follows: no variation in the MN, no MN tumor or tumor-like lesion, no trauma, no history of diabetes, rheumatoid arthritis, pregnancy, etc. In addition, all patients with CTS were confirmed by surgical intervention, and none of the control participants had NCS results.

### CTS Diagnosis

One experienced hand microsurgeon, with 11 years of hand surgery experience, evaluated patients who reported numbness or pain in the palm according to the 2016 American Academy of Orthopedic Surgeons (AAOS) Guidelines (18). Patients who reported neuropathy symptoms underwent further NCS and US examinations.

### NCS Examination

The NCS examinations were performed by an experienced neurologist, with 15 years of NCS experience, using a standard electromyography system (Natus Keypoint 9033A07). All inspections were performed at ambient temperature (25°C). The skin surface temperature in all cases was maintained at 33–35°C. All patients underwent NCS examinations according to the protocol recommended by the American Academy of Emergency Medicine (AAEM) (19). We measured the distal sensory nerve conduction velocity (SCV), motor nerve conduction velocity, distal and proximal motor latency, as well as the compound muscle action potential and sensory nerve action potential of the MN. The severity of NCS was classified as mild, moderate, or severe, and extreme according to a modified scoring system (20). Mild CTS was defined as a minimal abnormality in the segmental or controlled trials, moderate as abnormalities in the finger-wrist SCV or distal motor latency, severe as the lack of sensory response, abnormal distal motor latency, and reduced motor response, and extreme as the lack of movement and sensory responses.

### US and SWE Technology

Ultrasound and SWE examinations were performed using a 15–4 MHz linear array transducer (Supersonic, AIXplorer, France). The MN and TCL of all subjects were examined by a sonologist with 8 years of experience in musculoskeletal ultrasound (MSK-US) and elastography. The bilateral MNs were examined for each participant. The sonologist was blinded to the patient's clinical history and NCS results. The interval between the MSK-US and NCS was 1 week. For the examination, each participant sat opposite the sonographer and placed their hand on the examination table. The arm was naturally extended with the palm facing upward unless special circumstances required alternative positions, and the fingers were maintained in a relaxed semi-flexed position. To avoid compression of the MN, the operator gently placed the transducer on the skin with sufficient gel and kept the transducer stationary during the acquisition process. There were not any measurements made at the time of the US study.

#### Grayscale US Imaging

The US focus of elevation was placed at the level of the MN or TCL. The cross-section of the MN and TCL was imaged at the proximal carpal tunnel (PCT) (scaphoid-pisiform level) and distal carpal tunnel (DCT) (trapezium-hook level) along the short axis of the wrist. The TCL was characterized by a strip of high-low-high echoes located in the superficial square of the MN and was connected with the scaphoid-pisiform and trapezium-hook. The images were moderately enlarged using digital zoom until the MN and TCL were clearly identified, and they were then saved to the machine for later measurements.

#### SWE Imaging

The US focus of elevation was placed at the TCL level at the PCT and DCT. SWE imaging was then initiated and simultaneously displayed the elastic and grayscale images using a default scale (>600 kPa). SWE displays the tissue stiffness on a color scale ranging from blue to red, indicating low to high stiffness. To reduce interference from anisotropy, we selected a target region of the TCL perpendicular to the transducer. Six SWE images (three of the PCT and three of the DCT) were obtained using three penetration modes (Pen, Res, and Gen). The color of the TCL was displayed steadily for more than 3 s before freezing and capturing. All SWE images were saved to the computer for later analysis.

### Image Interpretation

All images were interpreted by two sonologists (with 8 and 7 years of experience in MSK-US and elastography). These sonologists measured the CSA_MN_ and AP_MN_, as well as the thickness and stiffness of the TCL. The thenar muscle was considered atrophied when the thickness became thin and the echo became enhanced (21). Both sonologists were blinded to the patient's symptoms and NCS results, in addition to each other's US and SWE image measurements. All measured values were averaged across the two sonologists. More measurement details of MN and TCL are shown in Appendix Figure 1.

#### Measuring the CSA_MN_ and AP_MN_

The boundary of the MN was determined, excluding the hyper-echo of the epineurium and the surrounding space. The two sonologists obtained the CSA_MN_ (cm^2^) and AP_MN_ (cm) by tracing the hypoechoic boundary of the MN at the PCT and DCT, respectively, using the grayscale images. The AP_MN_ was defined as being perpendicular to the maximum left-right diameter of the MN.

#### Measuring TCL Thickness

The measured TCL thickness (cm) was equivalent to the center of the MN cross-section at the PCT and DCT in the grayscale image. The measuring mark is placed from the volar and dorsal boundaries of the TCL.

#### Measuring TCL Stiffness

Transverse carpal ligament stiffness was measured at the PCT and DCT in the SWE image. The region of interest (ROI) was located at the center of the MN cross section. The ROI size of all cases was fixed at 1 mm. The stiffness of the TCL was measured from six images (three of the PCT and three of the DCT). The Emean, Emin, and Emax (kPa) values of the TCL were obtained for each image.

### Statistical Analysis

Statistical analysis was performed using R Studio v1.1 (SAS Institute, Inc, Cary, USA). Metadisc v1.4 (Unit of Clinical Biostatistics Team of the Ramón y Cajal Hospital, Madrid, Spain) was used to construct forest plots, and Adobe Illustrator 2020 (Adobe Institute, USA) was used for figure aesthetics. We calculated the average of each measurement obtained from both sonologists for statistical analysis. A normality test was performed for each baseline characteristic. If a characteristic followed a normal distribution, it is represented as the mean ± standard deviation (SD). Otherwise, it is represented as the median and the interquartile range (IQR). The significance level was set at *p* < 0.05.

The intra-class correlation coefficient (ICC) was used to evaluate inter-observer consistency. ICC > 0.75 indicates high consistency (22).

The differences in the CSA_MN_ and AP_MN_ as well as the TCL thickness and stiffness were analyzed using a paired sample *t*-test or the Wilcoxon signed-rank sum test for normally and non-normally distributed variables, respectively. A two-sample *t*-test and the Mann–Whitney *U*-test were used to analyze the differences in CSA_MN_ and AP_MN_ as well as the TCL thickness and stiffness. For participants in the patient group, we also evaluated the difference between same metrics and NCS results and progress using a random block test.

To investigate the diagnostic performance of CTS, the CSA_MN_, AP_MN_, and TCL thickness and stiffness were evaluated by constructing receiver operating characteristic curve (ROC). The area under the curve (AUC), sensitivity, and specificity were then calculated using the maximum Youden index (23). We then computed the pooled sensitivity and specificity to create a forest plot.

We studied the severity of NCS in US and SWE by dividing the patient group into 3 subgroups according to the NCS results of the participants' positive hand. In addition, we performed a similar analysis on these 3 subgroups We then computed the pooled sensitivity and specificity to create a forest plot.

## Results

### Participant Characteristics

**Figure 1:**
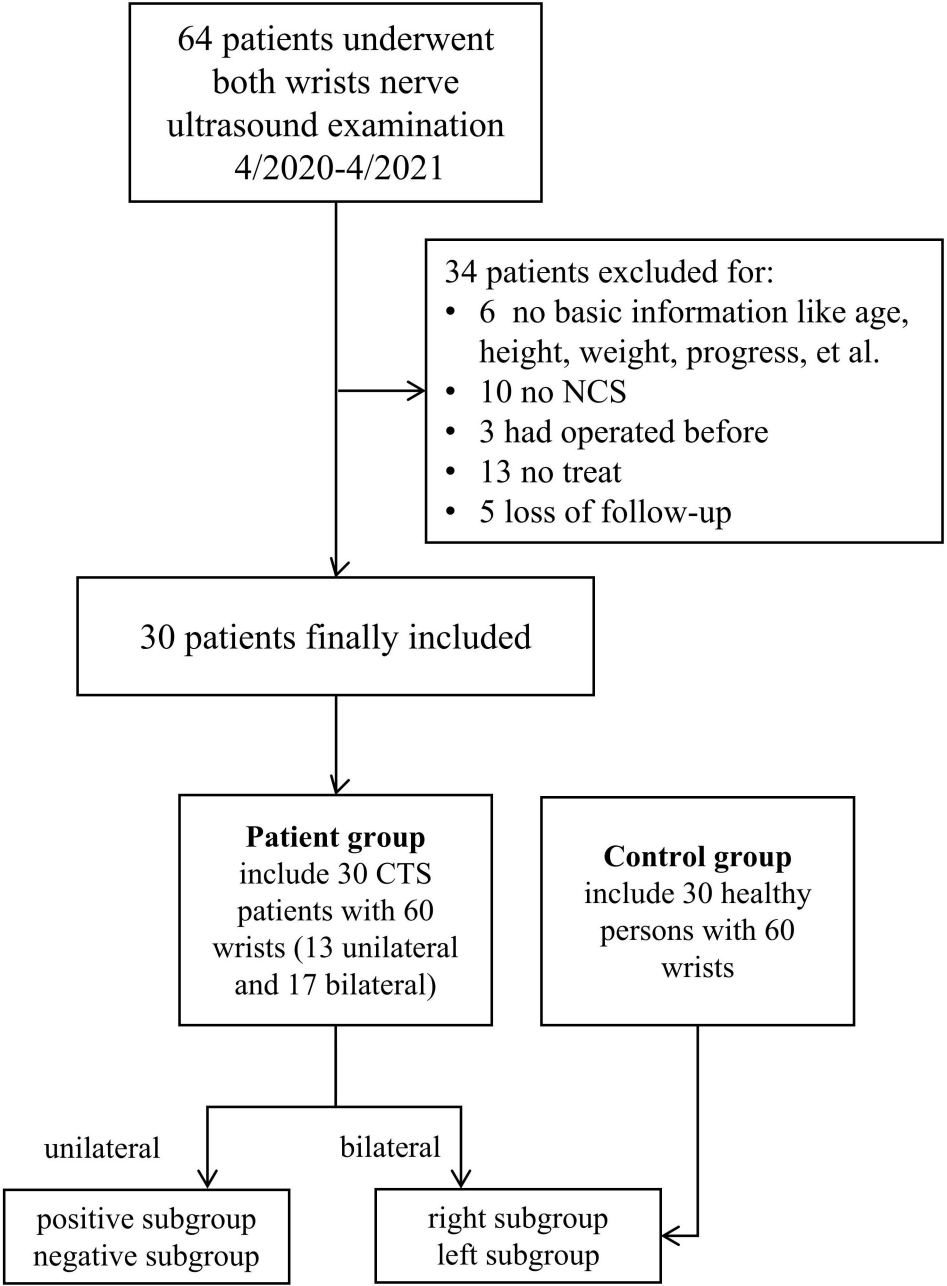
Study flowchart. NCS, nerve conduction study; CTS, carpal tunnel syndrome.

**Figure 2:**
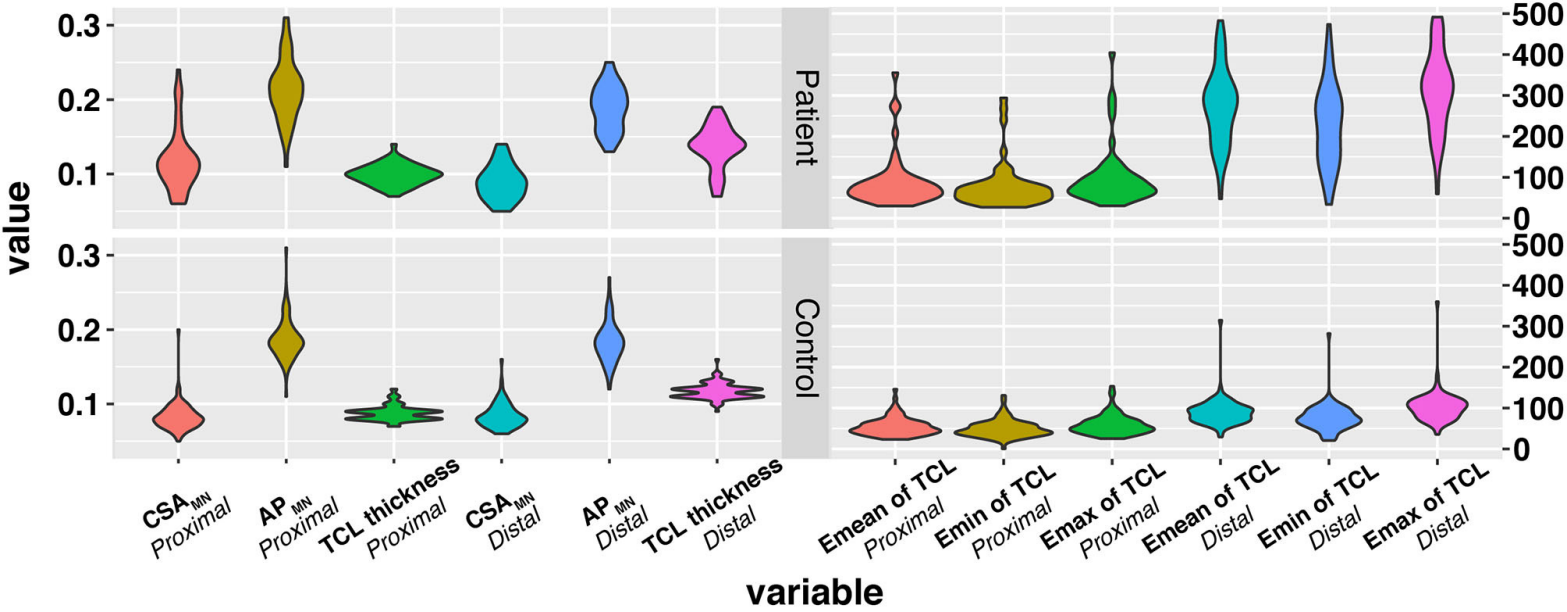
The distribution of variables in the three groups. The *x*-axis represents variables, and the *y*-axis represents the values of variables. The wider bar represents the more distributed data. TCL, transverse carpal ligament; CSA, cross sectional area; MN, median nerve; SWE, Shear wave elastography.

A total of 64 patients who underwent wrist nerve US examinations were initially included in this study (Figure 1). After applying the previously described exclusion criteria, the final study cohort consisted of 30 patients with CTS and 60 wrists (the patient group). In addition, thirty healthy volunteers and 60 wrists were included as the control group. The baseline characteristics of the 60 participants are presented in Table 1 and Appendix Table 1. The distributions of variables in both groups are shown in Figure 2.

**Table 1:** Baseline characteristics of the study participants.

**Variables**	**Total (*n* = 60)**	**Patient group (*n* = 30)**	**Control group (*n* = 30)**	***p***
Age, year, Mean ± SD	50.92 ± 10.67	52.23 ± 10.22	49.6 ± 11.12	0.344
Gender, female, n	60 [47]	30 (26)	30 (21)	0.21
Height, m, Median (Q1,Q3)	1.6 (1.57, 1.64)	1.58 (1.55, 1.63)	1.62 (1.6, 1.68)	0.009
Weight, kg, Median (Q1,Q3)	61 (57, 69.25)	60.5 (56, 68)	61 (58, 70)	0.579
BMI, Median (Q1,Q3)	23.84 (22.13, 25.32)	23.96 (22.55, 26.17)	23.49 (22.11, 24.95)	0.329
**Dominant hand**, ***n*** **(%)**				0.492
Left	2 (3)	0 (0)	2 (7)	
Right	58 (97)	30 (100)	28 (93)	
**Positive hand**, ***n*** **(%)**				<0.001
Right	8 (13)	8 (27)	0 (0)	
Left	5 (8)	5 (17)	0 (0)	
Both	17 (28)	17 (57)	0 (0)	
None	30 (50)	0 (0)	30 (100)	
**Right hand**				
Thenar (Amyotrophy)	60 (4)	30 (4)	30 (0)	0.112
Progress, year, *n* (%)				<0.001
0	35 (58)	5 (17)	30 (100)	
0~1	11 (18)	11 (37)	0 (0)	
1~2	7 (12)	7 (23)	0 (0)	
2~3	2 (3)	2 (7)	0 (0)	
>3	5 (8)	5 (17)	0 (0)	
NCS, *n* (%)				<0.001
Mild	1 (2)	1 (3)	0 (0)	
Moderate	16 (27)	16 (53)	0 (0)	
Severe	6 (10)	6 (20)	0 (0)	
Extreme	2 (3)	2 (7)	0 (0)	
Normal	35 (58)	5 (17)	30 (100)	
**Left hand**				
Thenar (Amyotrophy)	60 (3)	30 (3)	30 (0)	0.237
Progress, year, *n* (%)				<0.001
0	38 (63)	8 (27)	30 (100)	
0~1	12 (20)	12 (40)	0 (0)	
1~2	4 (7)	4 (13)	0 (0)	
2~3	1 (2)	1 (3)	0 (0)	
>3	5 (8)	5 (17)	0 (0)	
NCS, *n* (%)				<0.001
Mild	10 (17)	10 (33)	0 (0)	
Moderate	7 (12)	7 (23)	0 (0)	
Severe	5 (8)	5 (17)	0 (0)	
Extreme	1 (2)	1 (3)	0 (0)	
Normal	37 (62)	7 (23)	30 (100)	

*BMI, body mass index; NCS, nerve conduction study; SD, standard deviation. Parametric continuous variables are represented by mean ± SD and non-parametric variables are represented by median (Q1, Q3)*.

### Study Consistency

In this study, the measurements of the CSA_MN_, AP_MN_, and TCL thickness and stiffness were shown to exhibit satisfactory inter-observer reproducibility. More details on the results are shown in Appendix Text 1.

### Inter- and Intra-Group Differences

This study found that in the unilateral patient subgroup, the Emean, Emin, and Emax TCL values from the positive hand were significantly higher than the negative hand at the PCT and DCT (both, *p* < 0.05) (Table 2). For bilateral cases, patients were divided into right and left subgroups. The right hand CSA_MN_ at the PCT and the Emean, Emin, and Emax TCL values at the DCT were significantly higher than the left hand (all, *p* < 0.05) (Table 2). Further statistics of bilateral patient cases revealed that patients with two positive hands exhibited a significant difference in hand and NCS severity (all *p* < 0.01) (Appendix Table 2). The differences between the right and left hands of the control group and between the case and control groups are shown in Table 2, Appendix Table 3, and Appendix Text 2. The result of comparing the TCL stiffness and thickness in different gender are shown in Appendix Table 4 and Appendix Text 3.

**Table 2:** The MN (CSA and AP) and TCL (thickness and stiffness) measurements in the patient group with unilateral and bilateral positive hands, and the control group.

**Groups**		**Proximal carpal tunnel**, **Mean** **±SD, Median (Q1,Q3)**						**Distal carpal tunnel, Mean** **±SD, Median (Q1,Q3)**					
		**MN**		**TCL**				**MN**		**TCL**			
		**CSA, cm^**2**^**	**AP, cm**	**Thickness, cm**	**Emean, kPa**	**Emin, kPa**	**Emax, kPa**	**CSA, cm^**2**^**	**AP, cm**	**Thickness, cm**	**Emean, kPa**	**Emin, kPa**	**Emax, kPa**
**Patient group**													
Unilateral	Negative (*n* = 13)	0.09 (0.08, 0.09)	0.21 ± 0.04	0.09 (0.08, 0.1)	73.7 ± 32.87	60.1 ± 34.92	84.6 ± 35.18	0.08 (0.07, 0.1)	0.19± 0.04	0.12 (0.12, 0.13)	98.72± 72.06	58 (45.2, 76.7)	100.4 (67.7, 122.6)
	Positive (*n* = 13)	0.12 (0.09, 0.13)	0.22 ± 0.04	0.09 (0.09, 0.1)	74.8 ± 35.26	66.7 ± 29.59	82.9 ± 41.09	0.1 (0.07, 0.12)	0.2 ± 0.03	0.15 (0.14, 0.17)	295.75 ± 96.76	239 (161.1, 279.4)	340.3 (242.8, 460.7)
	P	<0.05	0.186	0.608	0.900	0.392	0.891	0.604	0.368	<0.001	<0.001	<0.001	<0.001
Bilateral	Left (*n* = 17)	0.1 (0.09, 0.11)	0.21 ± 0.04	0.1 (0.1, 0.11)	79.6 (60.1, 93.9)	71 (53.4, 78.8)	88.3 (65.8, 101.5)	0.08 ± 0.02	0.18 ± 0.03	0.14 (0.13, 0.16)	228.7 ± 84.2	186.9 ± 87.83	257.58 ± 90.63
	Right (*n* = 17)	0.12 (0.1, 0.15)	0.21 ± 0.04	0.1 (0.09, 0.11)	62.7 (50.7, 104.9)	56.3 (46.8, 87.9)	69.9 (58.6, 120.6)	0.09 ± 0.02	0.19± 0.03	0.14 (0.14, 0.16)	300.68 ±86.06	258.53 ± 103.58	340.25 ±77.85
	P	<0.05	0.942	0.311	0.392	0.459	0.818	0.071	0.548	0.103	<0.05	<0.05	<0.01
**Control group**													
Left (*n* = 30)		0.08 (0.07, 0.08)	0.18 (0.17, 0.19)	0.08 (0.08, 0.09)	54.36 ± 17.69	47.95(39.27, 60.27)	58.06 ± 18.44	0.08 (0.07, 0.08)	0.18 (0.16, 0.19)	0.12 (0.11, 0.12)	89.08 ± 20.46	77.28 ± 25.41	100.89 ± 21.76
Right (*n* = 30)		0.08 (0.07, 0.09)	0.18 (0.17, 0.19)	0.08 (0.08, 0.09)	46.46 ± 11.91	40.9 (35.9, 49.4)	49.92 ± 13.37	0.08 (0.08, 0.09)	0.18 (0.17, 0.2)	0.11 (0.11, 0.12)	89 ± 20.52	76.83 ± 21.5	100.81 ± 23.63
P		<0.05	0.312	0.225	<0.05	<0.05	<0.05	<0.05	0.059	0.321	0.987	0.918	0.988

*AP, anteroposterior diameter; E, Elastic Modulus; TCL, transverse carpal ligament; CSA, cross-sectional area; MN, median nerve*.

*Parametric continuous variables are represented by mean ± SD and non-parametric variables are represented by median (Q1, Q3)*.

### CTS Diagnosis

**Figure 3:**
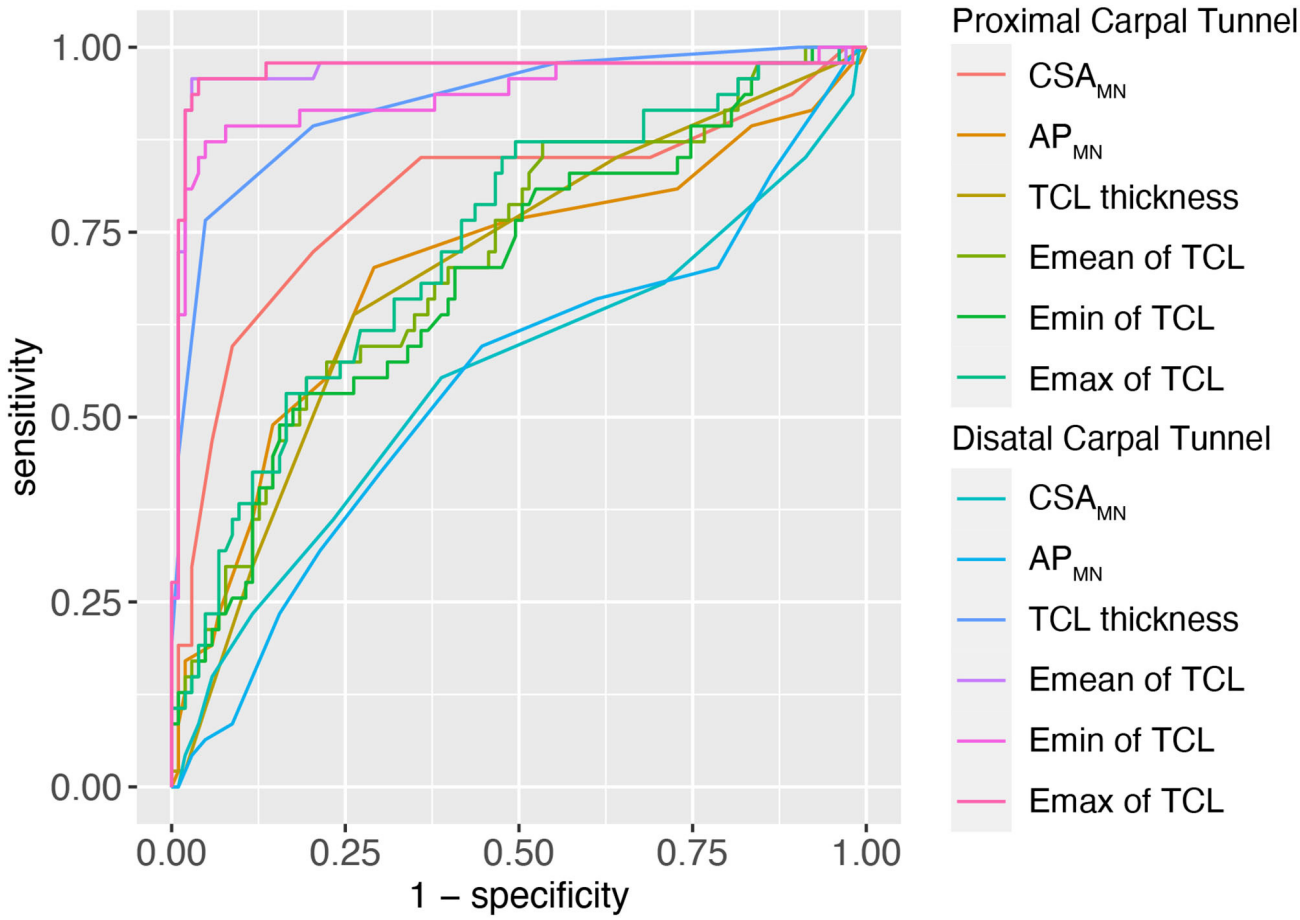
The ROC of MN (CSA and thickness) and TCL (thickness and stiffness) at proximal and distal carpal tunnel to diagnose CTS. TCL, transverse carpal ligament; CSA, cross sectional area; MN, median nerve; SWE, Shear wave elastography; ROC, receiver operating characteristic curve.

For the diagnosis of CTS, the AUC and cut-off values for the CSA_MN_, AP_MN_, TCL thickness and stiffness at the PCT and DCT are shown in Figure 3, Table 3. Among the multiple indicators, the AUC of the TCL thickness and stiffness at the DCT ranged between 0.816 and 0.967, and that of the CSA_MN_ (most commonly measured at the PCT) was 0.798.

**Table 3:** The AUC and cut-off values of the MN (CSA and AP) and TCL (thickness and stiffness) at the proximal and distal carpal tunnel (DCT).

**Variables**	**AUC**	**Cut-off**	**Sensitivity**	**Specificity**
**Proximal carpal tunnel**				
CSA_MN_, cm^2^	0.798	0.095	0.723	0.796
AP_MN_, cm	0.698	0.195	0.702	0.709
TCL, cm	0.698	0.095	0.638	0.738
Emean of TCL, kPa	0.713	68.85	0.553	0.806
Emin of TCL, kPa	0.692	63.40	0.532	0.816
Emax of TCL, kPa	0.734	53.90	0.872	0.505
**Distal carpal tunnel**				
CSA_MN_, cm^2^	0.554	0.085	0.553	0.612
AP_MN_, cm	0.546	0.185	0.553	0.596
TCL, cm	0.925	0.135	0.766	0.951
Emean of TCL, kPa	0.965	143.05	0.957	0.971
Emin of TCL, kPa	0.935	123.70	0.872	0.951
Emax of TCL, kPa	0.967	156.35	0.957	0.961

*AP, anteroposterior diameter; E, Elastic Modulus; TCL, transverse carpal ligament; CSA, cross sectional area; MN, median nerve; AUC, the area under the ROC curve*.

### NCS Severity

**Figure 4:**
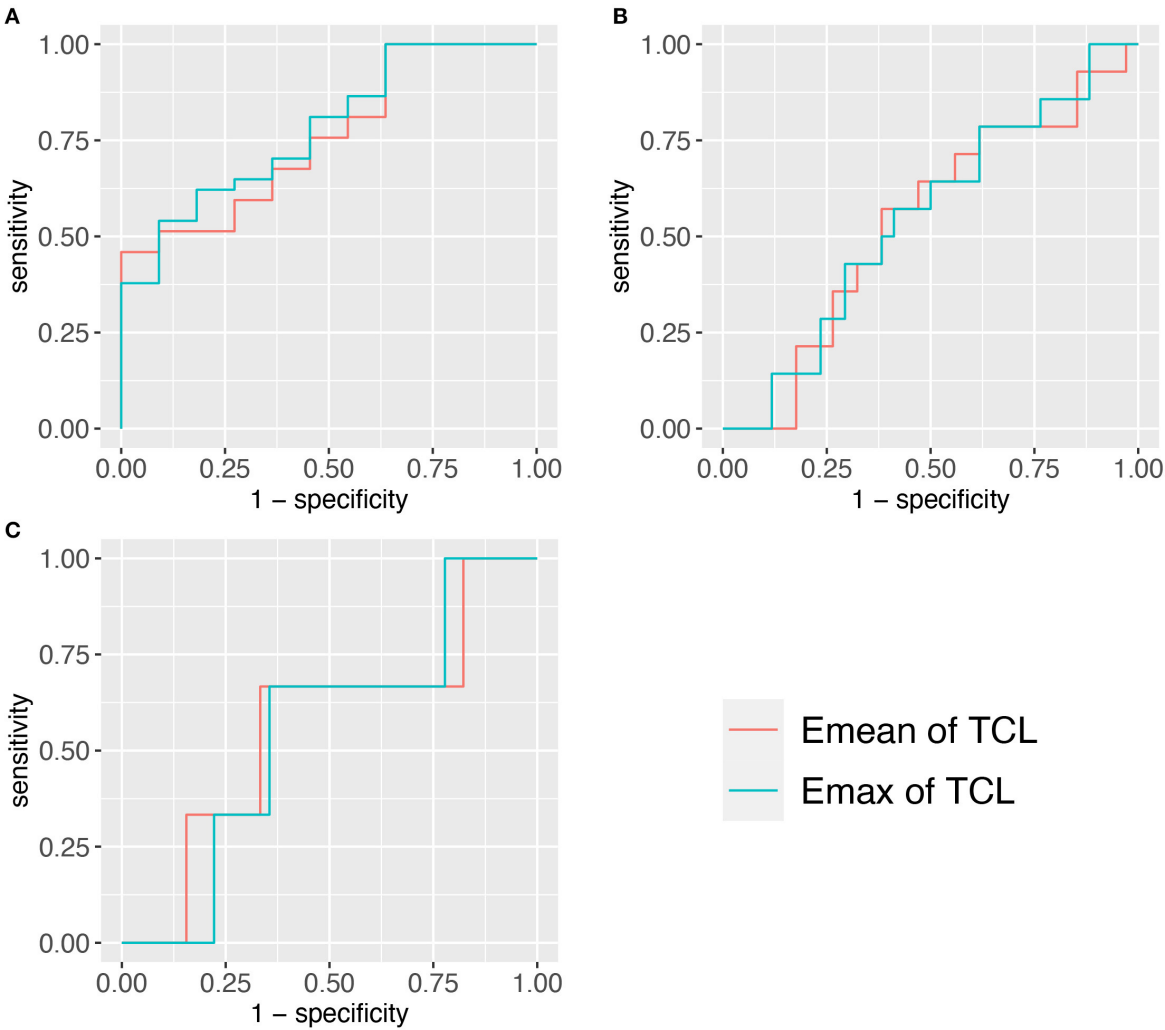
Three ROCs of TCL stiffness at the distal carpal tunnel (DCT) to diagnose different NCS severity. **(A)** <Moderate group. **(B)** <Severe group. **(C)** <Extreme group. TCL, transverse carpal ligament; SWE, shear wave elastography; ROC, receiver operating characteristic curve.

**Figure 5:**
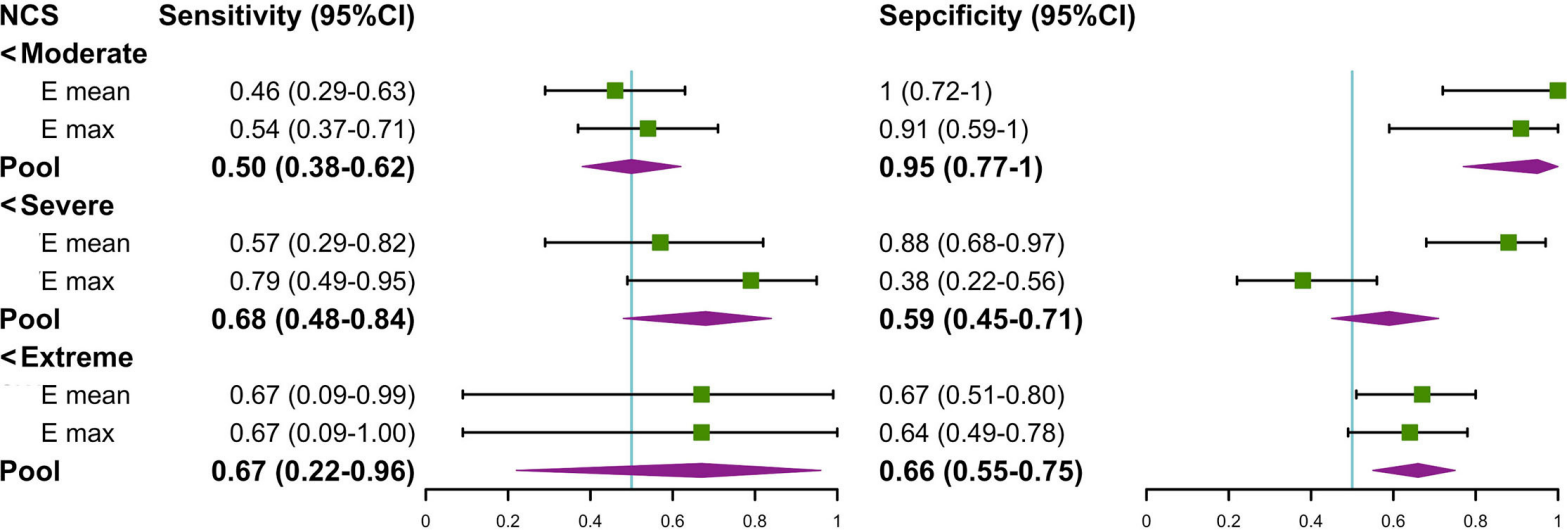
The forest plot about the pool sensitivity and pool specificity of the three groups. NCS, nerve conduction study; TCL, transverse carpal ligament; SWE, shear wave elastography.

We found that there was a significant difference in the Emean and Emax values of the TCL at the DCT and the severity of NCS for the positive hand in the patient group (both *p* < 0.05) (Table 4). We then divided the patient group into additional subgroups according to NCS severity (mild, moderate, severe, and extreme): (1) mild vs. moderate, severe, and extreme; (2) mild and moderate vs. severe and extreme; and (3) mild, moderate, and severe vs. extreme. The Emean and Emax values of the TCL were able to achieve an AUC of 0.757 and 0.779, respectively, for the 3 aforementioned comparisons (Figure 4, Table 5). The pooled sensitivity and specificity of these three analyses are shown in the Figure 5. There was no significant significance between the positive hands in the patient and progress (Appendix Table 5).

**Table 4:** Comparison of MN (CSA and AP), TCL (thickness and stiffness) and NCS severity in the positive hand in the patient group.

**Variables**	**Mild (*n* = 11)**	**Moderate (*n* = 23)**	**Severe (*n* = 11)**	**Extreme (*n* = 3)**	***p***
**Proximal carpal tunnel**					
CSA_MN_, cm^2^, Median (Q1,Q3)	0.1 (0.1, 0.11)	0.12 (0.09, 0.14)	0.11 (0.09, 0.12)	0.13 (0.12, 0.18)	0.11
AP_MN_, cm, Mean ± SD	0.2 ± 0.03	0.22 ± 0.05	0.22 ± 0.04	0.22 ± 0.02	0.614
TCL, cm, Median (Q1,Q3)	0.1 (0.1, 0.11)	0.1 (0.09, 0.1)	0.1 (0.1, 0.11)	0.1 (0.09, 0.11)	0.983
Emean of TCL, kPa, Median (Q1,Q3)	85.2 (61.4, 100.45)	61.7 (50.85, 80.25)	72.5 (60, 125.15)	76.8 (70.45, 90.85)	0.255
Emin of TCL, kPa, Median (Q1,Q3)	76.5 (54.4, 87.9)	55.8 (40.2, 72.85)	75.3 (54.25, 104.25)	70 (64.65, 78.95)	0.367
Emax of TCL, kPa, Median (Q1,Q3)	97.6 (67.85, 115.05)	66.3 (56.65, 90.7)	88.2 (66.25, 143.2)	83.8 (76.85, 102.2)	0.296
**Distal carpal tunnel**					
CSA_MN_, cm^2^, Mean ± SD	0.08 ± 0.02	0.09 ± 0.02	0.09 ± 0.02	0.08 ± 0.03	0.567
AP_MN_, cm, Mean ± SD	0.18 ± 0.03	0.19 ± 0.03	0.2 ± 0.03	0.19 ± 0.03	0.395
TCL, cm, Median (Q1,Q3)	0.16 (0.13, 0.16)	0.14 (0.14, 0.15)	0.14 (0.14, 0.15)	0.17 (0.16, 0.18)	0.131
Emean of TCL, kPa, Mean ± SD	209.23 ± 87.46	306.46 ± 87.24	270.54 ± 83.2	277.6 ± 87.64	<0.05
Emin of TCL, kPa, Mean ± SD	166.02 ± 88.51	257.86 ± 102.56	226.82 ± 80.47	250 ± 84.03	0.079
Emax of TCL, kPa, Mean ± SD	234.35 ± 94.52	351.88 ± 86.42	307.77 ± 91.96	314.7 ± 72.66	<0.01

*AP, anteroposterior diameter; E, Elastic Modulus; TCL, transverse carpal ligament; CSA, cross sectional area; MN, median nerve*.

*Parametric continuous variables are represented by mean ± SD and non-parametric variables are represented by median (Q1, Q3)*.

**Table 5:** The AUC and cut-off value for TCL stiffness at the DCT for diagnosing the severity of NCS.

**NCS**	**AUC**	
	**Emean of TCL**	**Emax of TCL**
<Moderate	0.757	0.779
<Severe	0.538	0.546
<Extreme	0.563	0.548

*E, Elastic Modulus; NCS, nerve conduction study; TCL, transverse carpal ligament; AUC, the area under the ROC curve*.

## Discussion

In this study, we found that the TCL of positive hands in the patient group were significantly thicker and stiffer than that of the negative hand in the patient group and control group. This was particularly true at the DCT and may be related to the severity of NCS.

The distal TCL is thicker and stronger than the proximal TCL because it exhibits a laminar configuration with a strong transverse bundle (24). The TCL is closely related to the activity of the thenar muscle, and is prone to distinct thickening and stiffening of the TCL in distal and radial locations (25, 26). Importantly, these structural changes can increase the chance of MN compression (27). We chose to measure the thickness and stiffness of the TCL, which corresponds to the center of the MN, because this landmark is closest to the MN. The MN is relatively radial at the DCT, so the TCL we measured is also radial. Due to this geometry, the TCL thickness and stiffness that we measured were significantly higher in patients with CTS. In this study, the thickness of the TCL at the DCT in positive hands in the patient group ranges from 0.11 to 0.18 cm, and in the control group ranged from 0.1 to 0.12 cm. These values were similar to other US-based *in vivo* studies, but thinner than some cadaver studies (15, 28, 29). The possible reason for this disparity is that the edge of thickened TCL is often blurred in US images because of the reconstruction in patients with CTS. This can easily lead to measurement errors even though the TCL thickness can be reliably imaged in healthy volunteers (14). Shen et al. (14) investigated the validity and reliability of US for measuring the TCL thickness by plotting a representative, digitalized outline of the TCL. However, this measurement method is difficult to be replicated in daily work. However, this highly reliable methodology can provide a training idea on automatic measurement of TCL thickness by artificial intelligence for future research.

Moreover, our study showed that the TCL thickness and stiffness are also related to the severity of NCS in the patient group. The results showed that the TCL stiffness exhibited the highest comprehensive value in discriminating between patients with mild and non-mild CTS cases (AUC: Emean of the TCL = 0.757 and Emax of the TCL = 0.779) and there is more clinical significance in differentially diagnosing mild CTS (the pooled sensitivity and specificity were 54 and 93%, respectively). Therefore, the TCL stiffness metric may be helpful in managing CTS patients with normal NCS and asymptomatic patients with MNW, who do not require treatment (6). The TCL is more easily pathologically thicken in MNW, which eventually leads to irreversible secondary axon loss and swelling in MN (30) even if the compression released. Some scholars have proposed that MNW is needed to be managed by NCS to take appropriate intervention measures (5). However, due to the many disadvantages of NCS (5), non-invasive methods, such as SWE examination are more conducive for disease management. In addition, we found that the TCL in the negative hand of the patient group was thicker and stiffer than that in the control group. This finding indicates that these patients can likely develop bilateral CTS and is an eye-opening reminder for at-risk patients to manage the use of wrists.

In previous studies, the diagnosis of CTS has been largely dependent on CSA_MN_ (7). The MN is often compressed in the carpal tunnel and causes swelling in the PCT or the inlet. However, nerve swelling is not limited to patients with CTS and can also occur in persons who suffer from peripheral neuropathy and even asymptomatic individuals (12). The AP can also assess the degree of nerve swelling, but is not as reliable as CSA (31). In our study, the AUC of the CSA in PCT was greater than that of the AP, but was lower than that of the TCL thickness and stiffness at the DCT.

We are not aware of other studies that have examined the difference in TCL stiffness between the left and right hand in healthy individuals. In this study, 28 volunteers were right-handed and 2 were left-handed, but the TCL stiffness of the left hand was significantly higher than the right. During follow-ups, we found that 43% (13/30) of the volunteers in the control group report that their left hand had a great daily load, such as in housework, dancing, and using a keyboard. The results may be related to the health of the volunteers, as it is possible to associate certain daily tasks with CTS (26).

Since the incidence of CTS is greater in women than in men, and less studies examined the differences between genders and TCL (thickness and stiffness). Our study showed that gender was not significantly related to TCL thickness and stiffness in the patient and control groups, which is consistent with previous studies (32, 33). However, female TCLs are more prone to strain (32, 33).

Based on the previous studies, we added some innovations. In a trial (15), the radial TCL region was significantly stiffer than the ulnar region using the ARFI in 8 patients with CTS. By contrast, we measured the closest site of TCL to the MN using SWE to obtain the most direct measurements. In addition, we performed more comprehensive comparisons between patients and healthy controls, and between different subgroups and found various correlations between TCL and NCS. Another creative study (29) investigated the TCL thickness and the associations with treatment methods. TCL thickness and stiffness were also included in our study, but the TCL thickness was not significantly related to NCS. It was likely due to the different standards for determining the CTS severity (symptoms vs. NCS). Nevertheless, we still believe that the TCL stiffness is a meaningful factor in the CTS management.

Although MN stiffness was not explored in this study, our team noted that some authors have considered the heterogeneity of the results regarding MN elastography (34, 35). They offset the errors caused by different weight and gender by the ratio of MN elasticity between the wrist and forearm. The ratio of thickness and stiffness of the proximal to the distal TCL can also be used to diagnose CTS, which is a follow-up study.

For the present, we found no studies on elasticity and surgical rehabilitation. However, some authors have conducted studies on rehabilitation after steroid injection (36, 37). The stiffness of MN and intracarpal tunnel contents decreases significantly after steroid injection, especially in patients who benefit from treatment.

There are some limitations of this study that should be noted. First, the sample size was relatively small. Second, we did not study the cases of patient with CTS that were caused by other etiologies due to many uncertainties. Third, the NCS results of the included cases were not evenly distributed across the different severity levels (extreme cases were rare). In addition, we did not study the elasticity of the MN because it is difficult to reliably measure this value at the DCT. Finally, there was a lack of patients suffering from multiple modes of MN compression. In addition, no US gel pad or fixating device was used during image acquisition.

## Conclusion

In this study, TCL thickness and stiffness at DCT are more effective than CSA_MN_ and AP_MN_ in the diagnosis of CTS, and it can also evaluate the severity. It can detect the course of median neuropathy and guide the surgical method.

## Data Availability Statement

The raw data supporting the conclusions of this article will be made available by the authors, without undue reservation.

## Ethics Statement

The studies involving human participants were reviewed and approved by Ethics Committee of Shenzhen People's Hospital. The patients/participants provided their written informed consent to participate in this study.

## Author Contributions

HW, FD, and JX contributed to the study concepts and designed the study. HW, ZD, and WL contributed to all the grayscale US and SWE examination. HW and KY contributed to the data analysis and interpreted the data. XC and ZL contributed to the NCS. KY contributed to the statistical analysis. HW prepared the manuscript. All authors contributed to the article and approved the submitted version.

## Conflict of Interest

The authors declare that the research was conducted in the absence of any commercial or financial relationships that could be construed as a potential conflict of interest.

## Publisher's Note

All claims expressed in this article are solely those of the authors and do not necessarily represent those of their affiliated organizations, or those of the publisher, the editors and the reviewers. Any product that may be evaluated in this article, or claim that may be made by its manufacturer, is not guaranteed or endorsed by the publisher.
