# Supplementary material for: Evaluation of the Transverse Carpal Ligament in Carpal Tunnel Syndrome by Shear Wave Elastography: A Non-Invasive Approach of Diagnosis and Management
Source: Front Neurol. 2022 Jul 1;13:901104. doi: 10.3389/fneur.2022.901104 (PMC9283864; doi:10.3389/fneur.2022.901104)
Supplement: Supplementary file 1 [file Data_Sheet_1.pdf]

Appendix Tab. 1: The baseline MN (CSA and AP) and TCL (thickness and stiffness) values in the patient and control groups.

Variables	Total (n = 75)	Patient group (n = 30)	Control group (n = 30)	p
Right hand				
Proximal carpal tunnel				
CSA_{MN}, cm2, Median (Q1, Q3)	0.09 (0.08, 0.12)	0.12 (0.09, 0.13)	0.08 (0.07, 0.09)	< 0.001
AP_{MN}, cm, Median (Q1, Q3)	0.19 (0.18, 0.22)	0.21 (0.19, 0.23)	0.18 (0.17, 0.19)	< 0.001
TCL thickness, cm, Median (Q1, Q3)	0.09 (0.08, 0.1)	0.1 (0.09, 0.11)	0.08 (0.08, 0.09)	< 0.001
Emean of TCL, kPa, Median (Q1, Q3)	52.3 (41.55, 70.27)	66.7 (51.42, 90.72)	44.6 (40.73, 56.1)	< 0.001
Emin of TCL, kPa, Median (Q1, Q3)	47.25 (39.08, 63.83)	62.65 (45.3, 85.15)	40.9 (35.9, 49.4)	< 0.001
Emax of TCL, kPa, Median (Q1, Q3)	58.35 (45.4, 74.95)	71.9 (57.7, 103.05)	47.9 (42.43, 59.52)	< 0.001
Distal carpal tunnel				
CSA_{MN}, cm2, Median (Q1, Q3)	0.09 (0.08, 0.1)	0.09 (0.08, 0.11)	0.08 (0.08, 0.09)	0.097
AP_{MN}, cm, Mean ± SD	0.19 ± 0.03	0.2 ± 0.03	0.18 ± 0.03	0.157
TCL thickness, cm, Median (Q1, Q3)	0.12 (0.11, 0.14)	0.14 (0.14, 0.16)	0.11 (0.11, 0.12)	< 0.001
Emean of TCL, kPa, Median (Q1, Q3)	111.85 (87.38, 272.1)	275.7 (183.78, 326.35)	95.1 (75.2, 101.13)	< 0.001
Emin of TCL, kPa, Median (Q1, Q3)	97.4 (69.55, 238.85)	238.9 (133.95, 294.25)	76.7 (60.25, 91.38)	< 0.001
Emax of TCL, kPa, Median (Q1, Q3)	124.8 (103.38, 316.22)	316.65 (228.62, 382.57)	108 (88.7, 117.8)	< 0.001
Left hand				
Proximal carpal tunnel				
CSA_{MN}, cm2, Median (Q1, Q3)	0.08 (0.07, 0.1)	0.1 (0.09, 0.11)	0.08 (0.07, 0.08)	< 0.001
AP_{MN}, cm, Median (Q1, Q3)	0.19 (0.17, 0.21)	0.21 (0.19, 0.23)	0.18 (0.17, 0.19)	< 0.001
TCL thickness, cm, Median (Q1, Q3)	0.09 (0.08, 0.1)	0.1 (0.09, 0.1)	0.08 (0.08, 0.09)	< 0.001
Emean of TCL, kPa, Median (Q1, Q3)	63.25 (46.6, 79.88)	73.9 (59.5, 92.93)	51.65 (42.45, 64.33)	0.001

Emin of TCL, kPa, Mean ± SD	58.02 ± 25.14	65.06 ± 29.63	50.97 ± 17.48	0.029
Emax of TCL, kPa, Median (Q1, Q3)	69 (52.58, 88.05)	81.8 (64.98, 101.22)	55.1 (46.33, 70.42)	< 0.001
Distal carpal tunnel				
CSA_{MN}, cm2, Median (Q1, Q3)	0.08 (0.07, 0.09)	0.08 (0.07, 0.1)	0.08 (0.07, 0.08)	0.85
AP_{MN}, cm, Mean ± SD	0.18 ± 0.02	0.18 ± 0.03	0.18 ± 0.02	0.224
TCL thickness, cm, Median (Q1, Q3)	0.12 (0.11, 0.14)	0.14 (0.12, 0.15)	0.12 (0.11, 0.12)	< 0.001
Emean of TCL, kPa, Median (Q1, Q3)	101.7 (74.2, 229.55)	230.1 (108, 299.42)	87.8 (71.5, 99.33)	< 0.001
Emin of TCL, kPa, Median (Q1, Q3)	91.35 (65.02, 158.52)	168.05 (72.35, 273.32)	76.3 (62.1, 95.62)	0.001
Emax of TCL, kPa, Median (Q1, Q3)	120.25 (94.33, 244.15)	245.5 (134.88, 337.72)	102.1 (83.58, 113.97)	< 0.001

Abbreviations: AP, anteroposterior diameter; E, Elastic Modulus; MN, median nerve; TCL, transverse carpal ligament; CSA, cross sectional area

Note: Parametric continuous variables are represented by mean ± SD and non-parametric variables are represented by median (Q1, Q3).

Appendix Tab. 2: The comparison of the bilateral positive hand and NCS in the patient group.

Variables	Left (n = 17)	Right (n = 17)	p
NCS, n (%)			<0.01
Mild	10 (59)	1 (6)	
Moderate	5 (29)	9 (53)	
Severe	2 (12)	6 (35)	
Extreme	0 (0)	1 (6)	

Abbreviations: NCS, nerve conduction study

Appendix Tab. 3: The comparison of the MN (CSA and AP) and TCL (thickness and stiffness) for intra-group and intra-subgroup comparisons.

Groups	Proximal carpal tunnel Mean ± SD, Median (Q1,Q3)						Distal carpal tunnel Mean ± SD, Median (Q1,Q3)					
	CSA_{MN}, cm^2	AP_{MN}, cm	TCL, cm	Emean of TCL, kPa	Emin of TCL, kPa	Emax of TCL, kPa	CSA_{MN}, cm^2	AP_{MN}, cm	TCL, cm	Emean of TCL, kPa	Emin of TCL, kPa	Emax of TCL, kPa
Positive hand in Patient group (n = 47)	0.11 (0.09, 0.13)	0.21 (0.19, 0.23)	0.06 (0.05, 0.07)	72.5 (52.7, 92.45)	66 (47, 81.7)	79.5 (60.55, 103.15)	0.09 (0.07, 0.1)	0.19 (0.16, 0.21)	0.06 (0.05, 0.07)	282.9 (200.45, 318.3)	239 (148.55, 282.3)	315.8 (245.5, 363.7)
Control group (n = 60)	0.08 (0.07, 0.09)	0.18 (0.17, 0.19)	0.05 (0.05, 0.06)	47.35 (40.77, 60.65)	43.2 (37.72, 56.12)	52.25 (42.93, 65.03)	0.08 (0.07, 0.09)	0.18 (0.17, 0.19)	0.05 (0.05, 0.06)	89.9 (73.33, 100.4)	76.3 (61.18, 95.2)	102.85 (83.72, 117.4)
p	< 0.001	< 0.001	< 0.001	< 0.001	< 0.001	< 0.001	0.12	0.093	< 0.001	< 0.001	< 0.001	< 0.001
Negative hand in Patient group (n = 13)	0.09 (0.08, 0.09)	0.2 (0.19, 0.23)	0.07 (0.06, 0.07)	68.4 (51.9, 89.9)	61.2 (39.6, 79.5)	83.8 (53.7, 97.8)	0.08 (0.07, 0.1)	0.18 (0.16, 0.22)	0.07 (0.06, 0.07)	71.3 (60.3, 108)	58 (45.2, 76.7)	100.4 (67.7, 122.6)
Control group (n = 60)	0.08 (0.07, 0.09)	0.18 (0.17, 0.19)	0.05 (0.05, 0.06)	47.35 (40.77, 60.65)	43.2 (37.72, 56.12)	52.25 (42.93, 65.03)	0.08 (0.07, 0.09)	0.18 (0.17, 0.19)	0.05 (0.05, 0.06)	89.9 (73.33, 100.4)	76.3 (61.18, 95.2)	102.85 (83.72, 117.4)
p	0.162	< 0.001	< 0.001	< 0.01	0.102	< 0.01	0.929	0.398	< 0.001	0.498	0.077	0.937

Abbreviations: AP, anteroposterior diameter; E, Elastic Modulus; TCL, transverse carpal ligament; CSA, cross section area; MN, median nerve; AP, Anteroposterior diameter

Note: Parametric continuous variables are represented by mean ± SD and non-parametric variables are represented by median (Q1, Q3).

Appendix Tab. 4: The comparison of the TCL (thickness and stiffness) in different gender in patient group and control group.

**Patient group**	Total (n = 60)	Female (n = 52)	M (n = 8)	p
TCL thickness, cm, Median (Q1, Q3)	0.1 (0.09, 0.1)	0.1 (0.09, 0.1)	0.1 (0.09, 0.11)	0.42
Emean of TCL, kPa, Median (Q1, Q3)	71.2 (52.43, 91.72)	71.2 (52.75, 91.72)	73.45 (48.97, 93.33)	1
Emin of TCL, kPa, Median (Q1, Q3)	63.8 (46.3, 81.1)	63.8 (47.27, 79.75)	57.9 (36.88, 86.43)	0.7
Emax of TCL, kPa, Median (Q1, Q3)	79.65 (60.02, 102.33)	79.65 (60.02, 102.33)	82.85 (57.08, 101.92)	0.97
TCL thickness, cm, Median (Q1, Q3)	0.14 (0.13, 0.15)	0.14 (0.13, 0.15)	0.14 (0.13, 0.16)	0.65
Emean of TCL, kPa, Mean ± SD	235.46 ± 114.24	237.78 ± 111.44	220.35 ± 138.65	0.74
Emin of TCL, kPa, Mean ± SD	195.05 ± 111.96	196 ± 108.78	188.88 ± 139.22	0.89
Emax of TCL, kPa, Mean ± SD	269.72 ± 124.14	272.27 ± 122.12	253.14 ± 144.48	0.73
**Control group**	Total (n = 60)	F (n = 42)	M (n = 18)	p
TCL thickness, cm, Median (Q1, Q3)	0.08 (0.08, 0.09)	0.08 (0.08, 0.09)	0.09 (0.08, 0.09)	0.08
Emean of TCL, kPa, Mean ± SD	50.41 ± 15.47	50.85 ± 15.48	49.39 ± 15.85	0.74
Emin of TCL, kPa, Median (Q1, Q3)	43.2 (37.72, 56.12)	43.3 (36.58, 59.85)	42.15 (38.97, 55.48)	0.63
Emax of TCL, kPa, Mean ± SD	53.99 ± 16.48	54.5 ± 16.47	52.79 ± 16.93	0.72
TCL thickness, cm, Median (Q1, Q3)	0.11 (0.11, 0.12)	0.12 (0.11, 0.12)	0.11 (0.11, 0.12)	0.62
Emean of TCL, kPa, Mean ± SD	89.04 ± 20.31	91.9 ± 19.67	82.37 ± 20.78	0.11
Emin of TCL, kPa, Mean ± SD	77.06 ± 23.34	79.17 ± 24.12	72.12 ± 21.24	0.27
Emax of TCL, kPa, Mean ± SD	100.85 ± 22.52	103.89 ± 20.59	93.76 ± 25.72	0.15

Abbreviations: E, Elastic Modulus; TCL, transverse carpal ligament.

Note: Parametric continuous variables are represented by mean ± SD and non-parametric variables are represented by median (Q1, Q3).

Appendix Tab. 5: The comparison of the MN (CSA and AP), TCL (thickness and stiffness) and progress in the positive hand in the patient group.

Variables	0 (n = 1)	0~1 (n = 23)	1~2 (n = 11)	2~3 (n = 3)	>3 (n = 10)	p
Proximal carpal tunnel						
CSA_{MN}, cm2, Median (Q1,Q3)	0.09 (0.09, 0.09)	0.11 (0.09, 0.13)	0.1 (0.1, 0.12)	0.12 (0.1, 0.12)	0.11 (0.1, 0.13)	0.888
AP_{MN}, cm, Mean ± SD	0.2 ± NA	0.22 ± 0.04	0.2 ± 0.04	0.21 ± 0.03	0.21 ± 0.05	0.687
TCL thickness, cm, Median (Q1,Q3)	0.09 (0.09, 0.09)	0.1 (0.09, 0.1)	0.11 (0.09, 0.11)	0.11 (0.09, 0.11)	0.1 (0.09, 0.1)	0.622
Emean of TCL, kPa, Median (Q1,Q3)	62.8 (62.8, 62.8)	78.5 (58.7, 92.45)	69.9 (51.75, 97.15)	103.6 (77.45, 188.4)	62.9 (43.52, 70.9)	0.382
Emin of TCL, kPa, Median (Q1,Q3)	58.5 (58.5, 58.5)	70.6 (53.25, 79.65)	68.3 (45.8, 91.5)	97 (69.45, 168.2)	57 (39.92, 62.95)	0.441
Emax of TCL, kPa, Median (Q1,Q3)	69.9 (69.9, 69.9)	88.3 (63.85, 103.15)	84.7 (61, 107.45)	114.3 (85.85, 206.55)	68.1 (48.98, 77.3)	0.351
Distal carpal tunnel						
CSA_{MN}, cm2, Mean ± SD	0.1 ± NA	0.09 ± 0.03	0.09 ± 0.02	0.1 ± 0.01	0.08 ± 0.02	0.823
AP_{MN}, cm, Mean ± SD	0.2 ± NA	0.19 ± 0.03	0.19 ± 0.03	0.18 ± 0.04	0.18 ± 0.03	0.95
TCL thickness, cm, Median (Q1,Q3)	0.13 (0.13, 0.13)	0.14 (0.14, 0.15)	0.16 (0.14, 0.17)	0.16 (0.14, 0.17)	0.14 (0.13, 0.15)	0.279
Emean of TCL, kPa, Mean ± SD	314.6 ± NA	294.87 ± 97.69	259.43 ± 71.2	256.2 ± 98.23	244.01 ± 102.86	0.605
Emin of TCL, kPa, Mean ± SD	282.1 ± NA	249.07 ± 108.74	227.6 ± 84.91	189.1 ± 82.45	192.04 ± 92.14	0.546
Emax of TCL, kPa, Mean ± SD	359.9 ± NA	328.22 ± 97.63	290.82 ± 71.69	339.37 ± 153.91	287.45 ± 116.38	0.708

Abbreviations: AP, anteroposterior diameter; E, Elastic Modulus; TCL, transverse carpal ligament; CSA, cross section area; MN, median nerve; SWE, Shear wave elastography; NA, not available

Note: Parametric continuous variables are represented by mean ± SD and non-parametric variables are represented by median (Q1, Q3)
