# Supplementary material for: Evaluation of the Transverse Carpal Ligament in Carpal Tunnel Syndrome by Shear Wave Elastography: A Non-Invasive Approach of Diagnosis and Management
Source: Front Neurol. 2022 Jul 1;13:901104. doi: 10.3389/fneur.2022.901104 (PMC9283864; doi:10.3389/fneur.2022.901104)
Supplement: Supplementary file 2 [file Data_Sheet_2.pdf]

Appendix Text 1:

Study Consistency

In this study, the measurements of the CSA_{MN}, AP_{MN} and TCL thickness and stiffness were shown to exhibit satisfactory inter-observer reproducibility. No statistically significant difference was found between the CSA_{MN}, AP_{MN} or TCL thickness and stiffness between the two evaluators. Specifically, the intra-observer ICC, as calculated based on the first evaluator, ranged from 0.804 to 0.856 for the various measurements.

Appendix Text 2:

In control group, the CSA_{MN} at the proximal and distal carpal tunnel and the Emean, Emin and Emax TCL values at the proximal carpal tunnel were significantly different between hands (all, $P < 0.05$). In addition, the TCL stiffness of the left hand was larger (stiffer) than that of the right ($P < 0.05$) (Tab. 2).

In addition, we found that the MN and TCL at the proximal carpel tunnel were larger and stiffer, respectively, for the positive hand in the patient (47 wrists) and control group (60 wrists) (both, $P < 0.001$) (Appendix Tab. 3). When comparing the negative hand in the patient (13 wrists) and control groups (60 wrists), the AP_{MN} and the thickness and stiffness of the TCL at the proximal carpel tunnel were larger in the patient group (both, $P < 0.001$) (Appendix Tab. 3).

Appendix Text 3:

In patient and control group, the TCL stiffness and thickness in different gender were not significantly different between hands (all, $P > 0.05$). (Appendix Tab. 4).
