# Supplementary material for: Evaluation of the Transverse Carpal Ligament in Carpal Tunnel Syndrome by Shear Wave Elastography: A Non-Invasive Approach of Diagnosis and Management
Source: Front Neurol. 2022 Jul 1;13:901104. doi: 10.3389/fneur.2022.901104 (PMC9283864; doi:10.3389/fneur.2022.901104)
Supplement: Supplementary file 3 [file Data_Sheet_3.pdf]

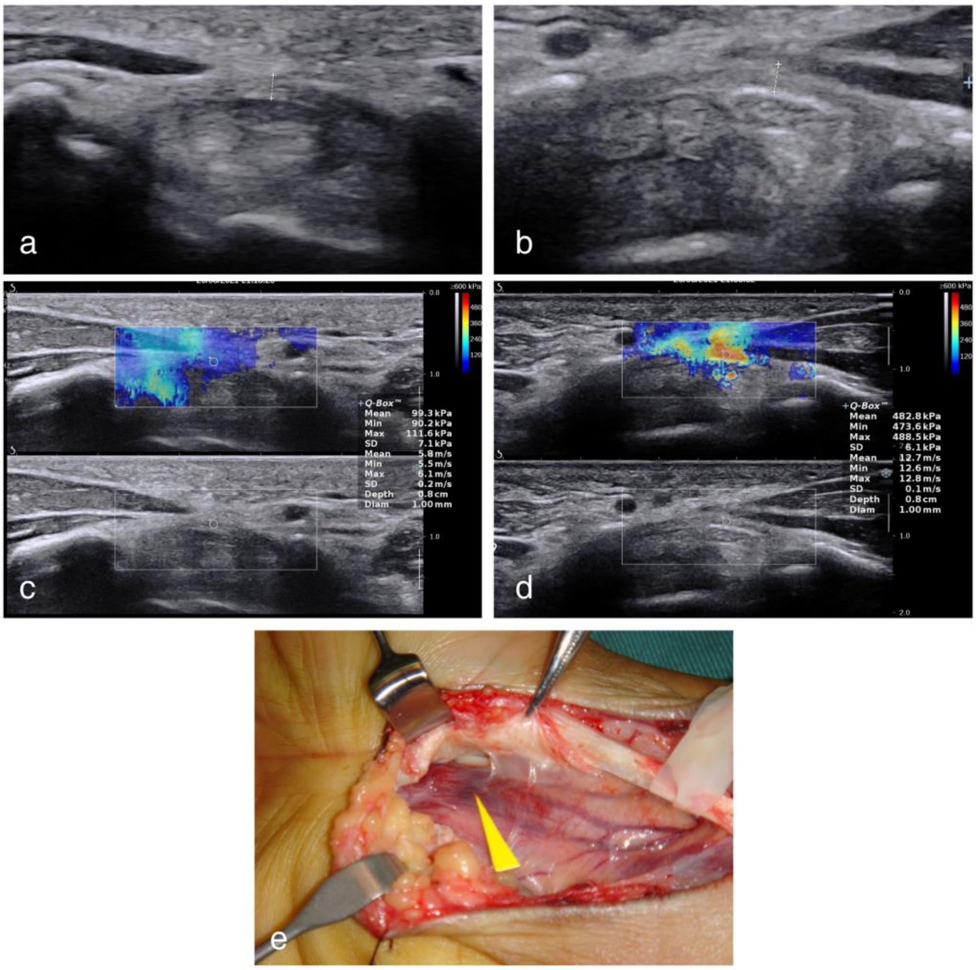

Appendix Fig. 1: The measurement of TCL thickness and stiffness at PCT.

A: TCL thickness in a healthy volunteer. It is 0.15cm;

B: TCL thickness of a CTS patient. It is 0.16cm;

C: TCL stiffness in a healthy volunteer. SWE mean: 99.3kPa, SWE min: 90.2kPa, SWE max: 111.6kPa;

D: TCL stiffness in a CTS patient. SWE mean: 482.8 kPa, SWE min: 473.6 kPa, SWE max: 488.5 kPa;

E: Surgery confirmed CTS.

Abbreviations: DCT, distal carpal tunnel; TCL, transverse carpal ligament; CTS, carpal tunnel syndrome

Note: +, between the two + represent TCL thickness in a and c; Circle, ROI of stiffness detection in b and d; Yellow arrow head: the position of median nerve be compressed
